# Postdiction: its implications on visual awareness, hindsight, and sense of agency

**DOI:** 10.3389/fpsyg.2014.00196

**Published:** 2014-03-31

**Authors:** Shinsuke Shimojo

**Affiliations:** Shimojo Psychophysics Laboratory, Division of Biology and Biological Engineering/Computation and Neural Systems, California Institute of TechnologyPasadena, CA, USA

**Keywords:** postdiction, flash lag, TMS, causality perception, hindsight, free will, sense of agency

## Abstract

There are a few postdictive perceptual phenomena known, in which a stimulus presented later seems causally to affect the percept of another stimulus presented earlier. While backward masking provides a classical example, the flash lag effect stimulates theorists with a variety of intriguing findings. The TMS-triggered scotoma together with “backward filling-in” of it offer a unique neuroscientific case. Findings suggest that various visual attributes are reorganized in a postdictive fashion to be consistent with each other, or to be consistent in a causality framework. In terms of the underlying mechanisms, four prototypical models have been considered: the “catch up,” the “reentry,” the “different pathway” and the “memory revision” models. By extending the list of postdictive phenomena to memory, sensory-motor and higher-level cognition, one may note that such a postdictive reconstruction may be a general principle of neural computation, ranging from milliseconds to months in a time scale, from local neuronal interactions to long-range connectivity, in the complex brain. The operational definition of the “postdictive phenomenon” can be applicable to such a wide range of sensory/cognitive effects across a wide range of time scale, even though the underlying neural mechanisms may vary across them. This has significant implications in interpreting “free will” and “sense of agency” in functional, psychophysical and neuroscientific terms.

## Introduction

This paper will review postdictive phenomena in perception and cognition, mainly from the author's own work with his collaborators but from some classical studies as well, to discuss the implications of these works. The first part of the paper will introduce a number of classical examples of “backward perceptual phenomena” (section Backward Perceptual Phenomena), as well as the flash-lag effect and its variations as more modern examples (section Flash-lag Effect, its Variations, and Object Updating). These phenomena will clearly suggest that there is a limited temporal time range (on an order of 100–200 ms) within which the processing of a stimulus presented later can affect the percept of another stimulus presented earlier. Starting from here, we will extend our review and discussion into several different directions. One unique contribution of ours is the TMS-triggered scotoma and the backward filling-in, which provide us with some insights into how cortical signals are dynamically reorganized (section TMS-Induced Scotoma, and Backward Filling-in). These may provide an empirical basis upon which to explore schematic prototypes of possible mechanisms (section Underlying Neural Mechanisms?). We will further extend our list of postdictive phenomena to (a) the memory and sensory consequences of voluntary movements (section Extending the “postdiction” Concept to the Memory and the Sensory Consequences of Voluntary Movements), to discuss neural and computational mechanisms further (section Neural and Computational Considerations), as well as (b) “hindsight bias” and cognitive reconstruction for consistency, at even longer time scales (section Hindsight Bias, and Cognitive Consistency). Whereas the underlying neural mechanisms in these cases may be different from the more sensory phenomena, the operational definition, the functional significance, and computational structure at an abstract level, of the “postdictive phenomenon” may still hold.

In the last few sections, we will further extend our discussion to Benjamin Libet's well-known claims, and the “free will” as endangered (section Libet's Claims, and the “free will” Endangered?). We will consider “sense of agency” as a postdictive attribution and an authentic illusion, as a solution to this contention (section “Sense of agency” as Postdictive Attribution and an Authentic Illusion).

This paper is not meant to be an inclusive overview of backward phenomena in general (in the context of prediction vs. postdiction to cope adaptively with neural delay, see Bachmann (2013) for a systematic overview.) Rather, it aims to focus on the variety of phenomena at a wide range of time scales, to discuss possible underlying mechanisms as well as philosophical/real-world implications.

## Backward perceptual phenomena

**Figure 1:**
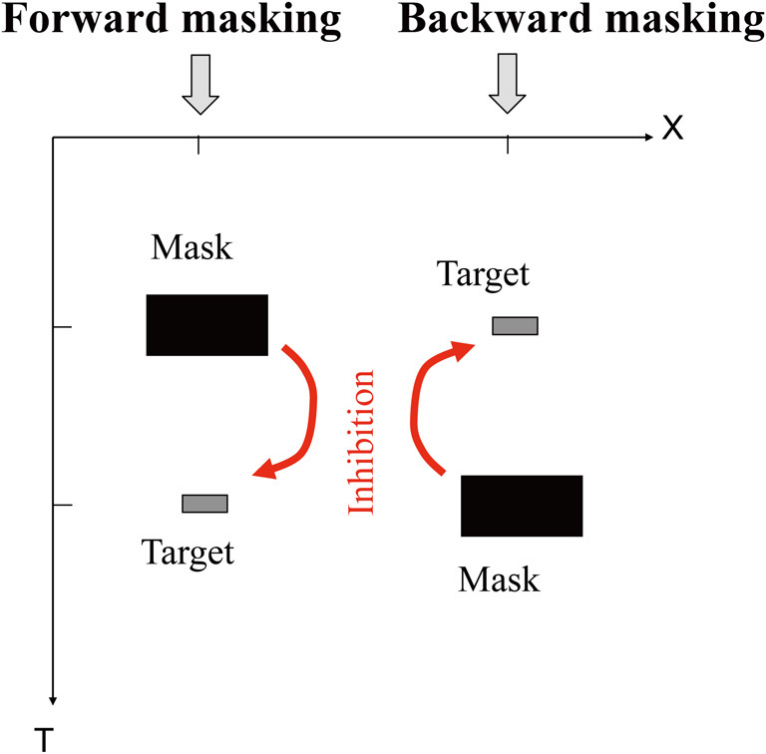
**Forward masking (left) and backward masking (right).** Space (X) × time (T) plot of the stimulus sequence and effects. Under appropriate conditions (<100 ms), a presentation of a mask prevents the target from being visible. The backward case, in particular, pauses a paradox in the framework of single-line, or feedforward (“Cartesian”) model of time.

There are a few classical perceptual phenomena in which a stimulus presented later seems causally to affect the percept of another stimulus presented earlier. (To avoid ambiguity, “seems to” above means “seems to scientists,” and “percept” means the “percept to the observer.”) We would like define “postdiction” or “postdictive perceptual phenomena” as such, throughout this paper. For example, a masking stimulus that is presented later can suppress the visibility of a target that is presented earlier in physical time (backward masking; see Figure 1).

**Figure 2:**
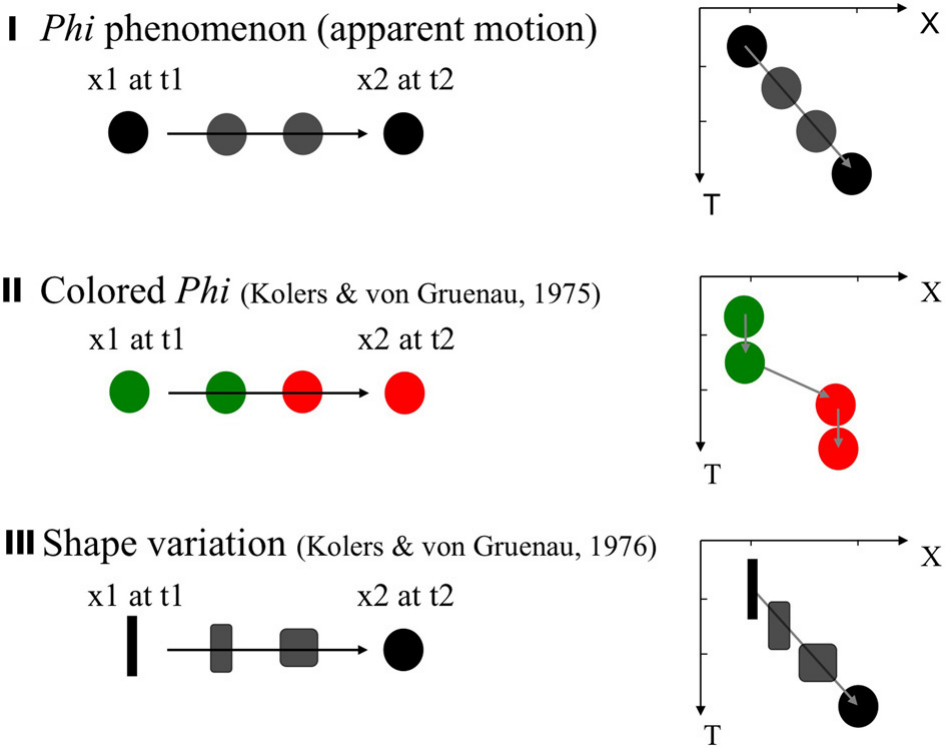
**Classical apparent motion, and variations.** The stimuli and percepts are illustrated on the left side, with the graphs on the right side shows space (X) × time (T) sequences of the stimuli and the percept. In the classical “phi” **(I)**, a pair of target separated within the optimal range of space and time distances lead to a percept of a smooth continuous visual motion. The colored phi **(II)** according to Kolers and von Grunau (1975, 1976) leads to an abrupt change of the color as well as the position. The shape variation **(III)** (Kolers and von Grunau, 1976) leads to a smooth impression of shape morphing, and seemingly works even under an one-shot presentation without prior knowledge or a cue.

Kolers and von Grunau (1975, 1976) examined the “color phi” situation. The stimuli are similar to those for the classical apparent motion (“phi”; Figure 2I), except that the two stimuli (snapshots) are colored differently (e.g., green and red). Their observer tended not to see a smooth change of colors, but instead saw an abrupt change of the color at one point, in the trajectory (Figure 2II). However, Kolers and von Grunua (1976) also reported that a shape version (with two distinctively different shapes in the two frames) works better (Figure 2III). In this case, a quick yet smooth morphing of contours/shape can be observed, which is clearly different from the color case. Moreover, this observation seems to hold even in the abrupt, one-shot presentation, as opposed to repeated presentations of the same sequence.

The one-shot observation case is more stringent and intriguing particularly when there is no clue or knowledge is given as to where and what is given in the second frame. In fact, even the most classical case of apparent motion should be considered postdictive under such a condition, as quite logically, the smooth trajectory of motion should be constructed only after the information about the second stimulus is given. Indeed, we have demonstrated that even in a condition in which the apparent motion can be leftward or rightward randomly across trials, the perception of apparent motion is no less obvious and/or smooth than the repeated case. Moreover, by adding an additional probe dot around the spatio-temporal trajectory of the apparent motion, we demonstrated that re-ordering of the temporal sequence of events occurs along with the spatio-temporal trajectory of motion (Nadasdy and Shimojo, 2010).

**Figure 3:**
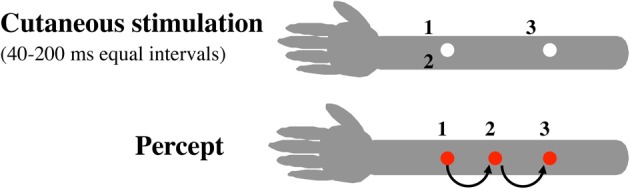
**Cutaneous rabbit (Geldard and Sherrick, 1972).** Numbers in the figure indicate the temporal sequence of tapping stimuli on the arm (with 40–200 ms equal intervals). A spatial interpolation occurs in this case—that is, the location of tap 2 is biased toward that of tap 3. Note that the event subsequent in time (3) causally affects the perceived location of the prior event (2).

Examples are not limited to vision. In the cutaneous modality, the most well-known form perhaps would be the “cutaneous rabbit” effect (Geldard and Sherrick, 1972; see Figure 3). The cutaneous stimulus sequence is composed as the following for this demonstration; three tap stimuli are presented sequentially on an arm with temporal intervals equal but locations different (e.g., the first and second stimuli at the same location, and with the third then jumps, as shown in Figure 3). In effect, the second tap is mislocalized in the direction of the third.

These and other backward perceptual phenomena are mostly established at phenomenological and experimental levels. They obviously impose a hard problem on any interpretations based on the “a one-directional, single arrow” analogy of time, along which only an earlier event causally affect another subsequent event. One may call this the “Newtonian” model (or “Cartesian theater” after Dennett and Kinsbourne (1992); see the same for a theoretical review of the postdictive phenomena). In neural processing terms, the model may be characterized as strictly feedforward. When one considers the mental time, however, this would be an unnecessarily strong, and inappropriate, analogy to the physical time. As will be suggested later (section “Sense of agency” as Postdictive Attribution and an Authentic Illusion), the perceptual sequence of two perceptual events (as the content of percept, in the Mind Time) should be strictly separated from the physical sequence of corresponding neural events (in the Brain Time: see Figure 6). In other words, the strict isomorphism is not guaranteed to hold in the microscopic temporal domain (as analogous to no direct isomorphism hold between spatial perception and spatial relationship of neural activity in the brain). We will revisit to detail this point later (section “Sense of agency” as Postdictive Attribution and an Authentic Illusion).

There is yet another line of perceptual phenomena which are closely related to the backward phenomena, and indeed yielded the concept of “postdiction” via debates concerning the underlying mechanisms—that is, the flash-lag effect and variations of it, as discussed next.

## Flash-lag effect, its variations, and object updating

**Figure 4:**
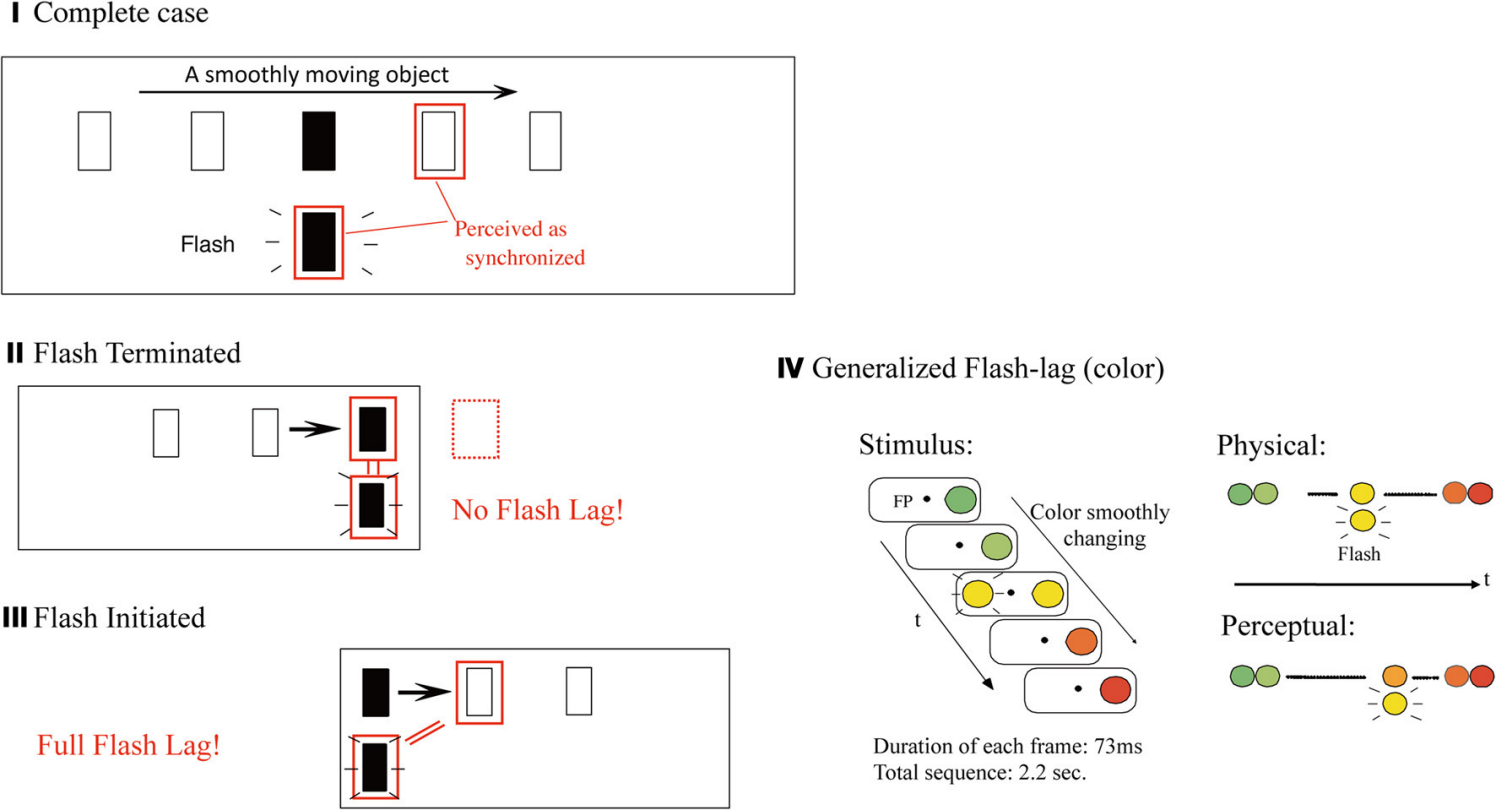
**Flash lag effect and its variations.** In the classical (or the “complete”) case by Nijhawan (1994) **(I)**, a flashed target appears to be lagged relative to a smoothly moving one. In the case of “flash terminated” where the motion trajectory after the presentation of the flash target is eliminated **(II)**, there is no flash-lag effect observed. Finally, in the case of “flash initiated” where the motion trajectory before the flash target is eliminated **(III)**, a qualitatively similar and nearly a full amount of the effect can be observed (Eagleman and Sejnowski, 2000; Nijhawan, 2008). This is unexpected from any account relying on the role of the prior trajectory (and extrapolation/expectation from it). **(IV)** Illustrates the “generalized flash lag” effect (Sheth et al., 2000). The sustained object did not move its position, but instead smoothly changing in one visual attribute such as color (or luminance, size, spatial randomness, etc.), and another object is briefly flashed with a color which matches the changing object's color at the moment. In the illustrated example, the flashed yellow is perceived simultaneously as the orange color of the color-changing object. (Modified from Sheth et al., 2000, Figure 1.)

Consider a smoothly moving object with yet another flashed object. Even when the flashed one is vertically aligned in its position with the moving object, the moving object tends to be mislocalized ahead in the direction of the motion (Figure 4I). This is called the “flash lag effect” (Nijhawan, 1994). The initial interpretation was that the brain predicts along the motion trajectory, to compensate its own neural processing delay by perceiving it ahead (but only for the moving stimulus, not for the flashed stimulus which is harder to predict). This was consistent with other circumstantial evidence that the brain compensates for its own delay (e.g., Changizi et al., 2008). However, a variety of other hypotheses/theories have been proposed to account for the effect, and none have been conclusive thus far (for a review, see Nijhawan, 2002).

What is critical in the current context is the following counter intuitive fact: the “flash terminated” case, where the moving and the flashed object disappear at the same time (Figure 4II), does not yield the effect (that is, the location of the flashed object is not mislocalized). The “flash initiated” case, on the other hand, where the two objects appear at the same time, with one continuing to move while the other disappears immediately, as “flashed” (Figure 4III), yields the effect (Nijhawan, 2008). Obviously, it is counterintuitive in any views of the effect based upon the predictability of the position of the moving target from its prior trajectory. To account for such a retrospective modulation of conscious visual perception, Eagleman and Sejnowski (2000) proposed a “postdiction” mechanism in which the percept attributed to the time of the flash is a function of events that occur in a timewindow of a maximum 80 milliseconds after the flash. Also note, with regard to the main theme of this paper, that they consider the postdictive process as a mechanism to yield visual awareness, or a conscious percept (beyond the mere operational definition of the “postdictive phenomena”; section Backward Perceptual Phenomena).

Figure 4IV illustrates “generalized flash lag” effect (Sheth et al., 2000). The sustained object did not move its position, but instead was smoothly changing in terms of one visual attribute such as color (or luminance, size, or spatial randomness, for instance), and another object is briefly flashed with a color which matches the changing object's color at that moment. In the example illustrated in the figure, the flashed yellow is perceived simultaneously as the orange color of the color-changing object. The effect is structurally similar to the classical flash-lag in the space/position domain; that is, a color of the changing object subsequent to the moment of presentation is perceived as simultaneous with the flashed. It is also critical to note the asymmetric pattern of results, similar to that in the classical flash lag effect—that is, the generalized flash lag tends to occur when the second half of a stimulus movie is presented (starting from the flash of the target; the “flash initiated”), but not when it is terminated there (the “flash terminated”).

It may be fair to say that there are some “non-postdictive” accounts proposed for the flash lag effect, and specifically the flash terminated case. For example, one may rely on the alleged extra neural delay (from the stimulus onset to the onset of conscious perception) of the suddenly-flashed object relative to the moving object (e.g., Whitney and Murakami, 1998). This account may be generalized to any sorts of smooth stream of an object representation with an abrupt onset of another object, thus possibly to the generalized flash lag effect. However, the mere fact of different neural delay may be somewhat dubious (see Moutoussis and Zeki, 1997; Nishida and Johnston, 2002). Moreover, the situation seems to be a bit more complicated, and other factors such as whether stimulation comes in stream or flashed plays a role (Bachmann, 2010, 2013; Bachmann et al., 2012).

Either way, the postdictive account of the flash lag effect, especially of the flash terminated case is worth mentioning here, for several reasons. First, it may be considered the original case of the term “postdiction” specifically employed to describe the retrospective modulation of visual awareness. Second, along with our strictly operational definition of the postdictive phenomena (section Backward Perceptual Phenomena), a physically subsequent event (of the moving object) affects the perceptual (spatio-temporal) relationship between it and another flashed object. Therefore, the neural delay accounts should be considered “non-postdictive” mechanisms which are still proposed to account for the postdictive (flash-lag) effect (operationally defined). Third, this is a rich perceptual phenomenon with a wide range of variations where a physical spatio-temporal sequence of visual stimuli leads to a percept of different sequence, thus providing ample opportunities to investigate underlying mechanisms of the phenomena postdictive.

One may still wonder what relationship the phenomena described so far have to the idea of “object updating” by James Enns et al. The object updating framework may be considered the closest to the idea of postdiction and related phenomena which are outlined here. A closer comparison may reveal similarity as well as the current implications beyond those of the object updating.

Object updating refers to the process whereby recently sampled information is integrated with an existing representation of a scene, resulting in an updated version (e.g., Lleras and Moore, 2003; Lleras and Enns, 2004; Moore and Enns, 2004). They argue that this theoretical framework provide a more comprehensible account for a variety of effects, such as the object substitution masking (especially the *Negative Compatibility Effect*, or the *NCE*), and the flash lag effect, etc.

The negative compatibility effect (NCE) is the surprising finding that visual targets that follow a brief prime stimulus and a mask can be *identified more rapidly when they are opposite* rather than identical to the prime. This was originally taken to reflect a competition between inhibitory unconscious processes and excitatory conscious processes (Klapp and Hinkley, 2002). However, Lleras and Moore (2003) offered an alternative account based on the object updating. If the perceptual processing interacts between the prime and the mask features, these seemingly neutral masks may, in fact, act as strong positive primes for the features that are not shared between prime and mask, they argue.

Likewise, the object updating may provide an alternative account especially for the classical, and some special variations of the flash lag effect (Nijhawan, 2008), where a smoothly moving object appears to be ahead in its trajectory, relative to a simultaneously flashed another object. The effect occurs when the moving object continues following the flash, but is eliminated if the object's motion path ends with the flash, as described above (the “flash terminated”). In the object updating framework, this may be interpreted as proving the necessity of updating the object representation after the flash. It seems to be consistent with the postdictive account of the effect, but with a somewhat different emphasis.

Whereas the object updating emphasizes the distinction between a representation of new object vs. that of the same object with feature changes, the postdictive construction view emphasizes that the content of conscious percept (e.g., the spatial alignment judgment of the two objects in this case) is a postdictive construct at an implicit level. The critical phenomenological observation here is that the updated representation is “experienced” as a percept, but “referred back” in time to the original moment of focus. It will be clearer especially in the case of the postdictive phenomena in a longer time scale (section Extending the “postdiction” Concept to the Memory and the Sensory Consequences of Voluntary Movements), but isomorphically true in nearly all the cases dealt in the current paper.

The object updating theory seems to be relatively limited to a short time range within several hundred ms or so, and to only a handful of visual effects, as mentioned above. The critical question raised in the section Extending the “postdiction” Concept to the Memory and the Sensory Consequences of Voluntary Movements and the subsequent sections will be whether the postdiction framework, while highly consistent with the object updating, will offer a more inclusive (or at least continuous) list of phenomena over-arching a much wider range of time scale, from teens of ms (at the level of sensation) to months (at the level of long-term memory and cognition).

## TMS-induced scotoma, and backward filling-in

Transcranial Magnetic Stimulation (TMS) is an intriguing technique which is used to stimulate or to suppress visual cortical activity, without stimulating the retina with a light. It is intriguing specifically in the current context because when using it, one may investigate how the direct manipulation (activation/suppression) of the visual neuronal activity can interact, and be integrated with the retinal signals.

**Figure 5:**
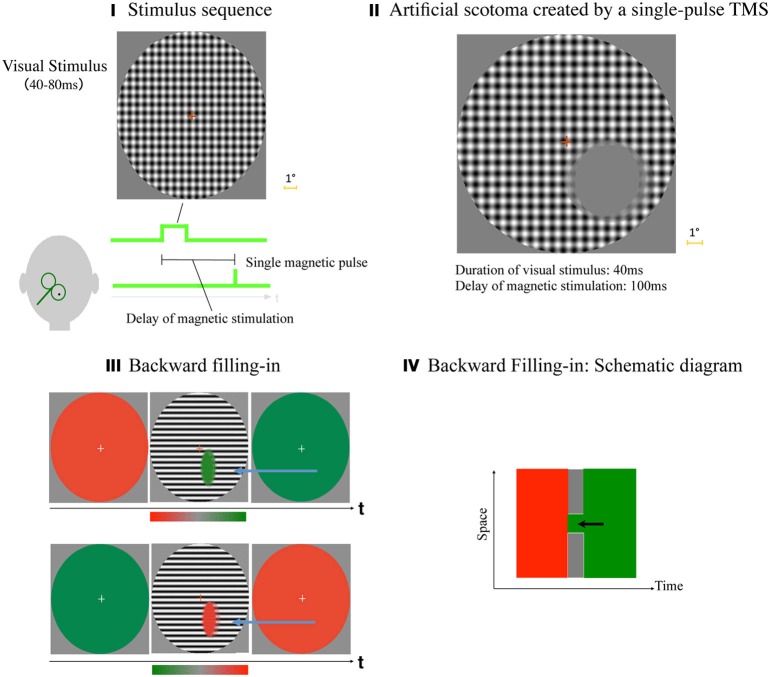
**TMS-induced scotoma, and backward filling-in. (I)** Stimulus sequence. After the participant fixated at a fixation point, a large-field patterned stimulus was presented for a brief time period (40–80 ms depending on the experiment), followed by a single-pulse TMS applied to the scalp over the primary (and possibly the secondary) visual cortex. The participant was asked to report the phenomenological size, shape and location of the TMS-induced scotoma by manipulating the mouse with a computer graphics software. **(II)** An example of actual data set. Results, graphics drawings of the scotoma in five trials within a participant with a fixed delay were superimposed, in order to show across-trial reliability of the effect. **(III)** Backward filling-in. The sequence of stimulus presentation, and also the result, i.e., averaged color chosen across the participants, were illustrated for “a red background (5 s) → BW stripes (80 ms) → a green background (5 s)” sequence (top row of the figure), and “a green background → BW stripes → a red background” sequence (bottom row). The colors filled in the scotoma in this figure are both what the participants have chosen on average. (Modified from Kamitani and Shimojo, 1999, Figure 5.) **(IV)** Backward filling-in: schematic diagram to summarize the finding in a Space × Time diagram. When local visual signals were suppressed creating a scotoma, the scotoma was filled-in with the color of the subsequent background, backwards in time (as indicated by the black arrow). In effect, then, the filled-in color and the surrounding striped pattern were perceived simultaneously, even though they were given to the retina subsequently in the physical time.

We demonstrated that an artificial and temporal scotoma can be created by a combination of a visual stimulus and a single-pulse TMS (Kamitani and Shimojo, 1999). In each trial, there was a fixation point on a gray background, and a large-field grid stimulus was presented briefly (40–80 ms). After a variable delay, a single-pulse TMS was applied to the occipital scalp (Figure 5I). When the delay of the magnetic stimulation was within 67–200 ms, the observer typically reported a scotoma, i.e., a gray homogenous patch in the hemi visual field contra-lateral to the TMS (Figure 5II). The phenomenology was qualitatively common and reliable across participants. We could even ask them to draw a gray-filled elliptic patch by adjusting its size via a mouse. Figure 5II shows an example of an actual data set obtained that way. The results in five trials within a participant with a fixed delay were superimposed, in order to show the across-trial reliability of the effect.

In a subsequent experiment, we maintained the stimulus sequences, but changed the color of the background: there was initially a red(green) background for 5 sec, then a black-and-white stripes for 80 ms., and finally green(red) background for 5 s (Figure 5III). (A two-dimensional grid was used in the first experiment, whereas stripes were used in this experiment. As a result, the scotoma was compressed along the orientation of the stripes, which is not essential given the current context.) With this design, we tried to address the following question—why did we perceive the gray-filled scotoma filled gray in the first experiment? Was it because all of the color selective neurons were equally suppressed by the TMS (hypothesis 1: the “broken color TV” hypothesis), or merely because the background in the corresponding retinal region was occupied by gray as a part of the preceding background (hypothesis 2: “forward filling-in” hypothesis)? The participant's task in this particular experiment was to report the filled color inside the scotoma by pointing and click on a continuous color scale showing a smooth transition from a pure gray to the most saturated red (green).

The results betrayed both of the hypotheses above, as shown in the figure (Figure 5III). The colors in the scotoma in the upper and the lower row of the figures were the actually selected color, averaged across the participants. Thus, when the subsequent background was green (the preceding was red; the upper row), a green-filled scotoma resulted. When the subsequent background was red (the lower), it then was red-filled. Thus, a sort of “backward” filling-in seemed to occur.

Figure 5IV schematically summarizes the result. When a local region of the topographical map of the visual field in the early visual cortices was suppressed by the TMS, the corresponding region in the grid/stripe pattern was perceived as a scotoma. The scotoma, however, was filled backward from the subsequent background color (indicated by the black arrow); thus, the stimuli presented only sequentially on the retina (i.e., the grid pattern and the subsequent background color) were perceived simultaneously in the particular spatial configuration (i.e., the elliptic scotoma in the large BW-patterned field). The filling-in is “backward” in this limited sense.

According to our informal observations, qualitatively identical results can be observed when we replaced the colored backgrounds with textured ones (although colors were the easiest to identify and thus to report). Therefore, the backward filling-in is a general phenomenon, not specific to color. When a part of the topographical representation was lost (by the TMS with a delay shorter than 200 ms), the visual cortex automatically utilizes the latest input in the particular region (the scotoma) at the moment and fills it in. This is consistent with our findings of the TMS-triggered replay of a visual stimulus (Wu and Shimojo, 2002, 2004; Halelamien et al., 2007; Vasudevan et al., 2009), indicating that content of a conscious percept is determined by the interplay of the retinal input and the internal state of the visual cortex at the moment.

Since this is a very special case with TMS, not with regular retinal inputs, it may not be appropriate to include it in the list of the “postdictive visual phenonena.” Indeed, one may account for the backward filling-in effect strictly relying on the instantaneous effect of the TMS on the visual cortex, as opposed to the neural conductance delay from the retina to the primary visual cortex, in the vicinity of 80–100 ms minimally. But even so, this may still be considered a special case of the “catch up,” as described as the first prototypical neural model of the postdiction mechanism in the next section Underlying Neural Mechanisms?. Moreover at the very phenomenological level, the background color (or pattern) in the scotoma area is perceived as “simultaneous” as the surround target pattern, which is qualitatively different from the temporal sequence of the visual stimuli. This is consistent with the operational definition of the postdictive phenomena. The TMS and retinal inputs are interactively compromised to yield a stable spatial percept (for instance, the shape of the scotoma is filled in and thus squeezed along the direction of background stripes; Kamitani and Shimojo, 1999), and this is reminiscent of the case of “smooth pursuit mislocalization” which will be described in section Pursuit Mislocalization, and Effects of the Spatial Context.

The set of findings with TMS allows us a glimpse into the dynamic process of integration to yield a postdictive effect at the early cortical levels within a 100–200 ms time window. Although in the previous examples of visual postdiction phenomena there was no direct stimulation/suppression of the visual cortical activity, a qualitatively similar process may operate during the dynamic reorganization of inputs. Overall, these findings indicate that dynamic, and at least partly postdictive processes are involved in the neural mechanisms yielding visual awareness, or a conscious percept.

Before moving on further to extend our list of postdictive phenomena to a more macro timescales, we would like to consider what prototypical neural/psychological mechanisms are conceivable as candidate underlying mechanisms (next section).

## Underlying neural mechanisms?

**Figure 6:**
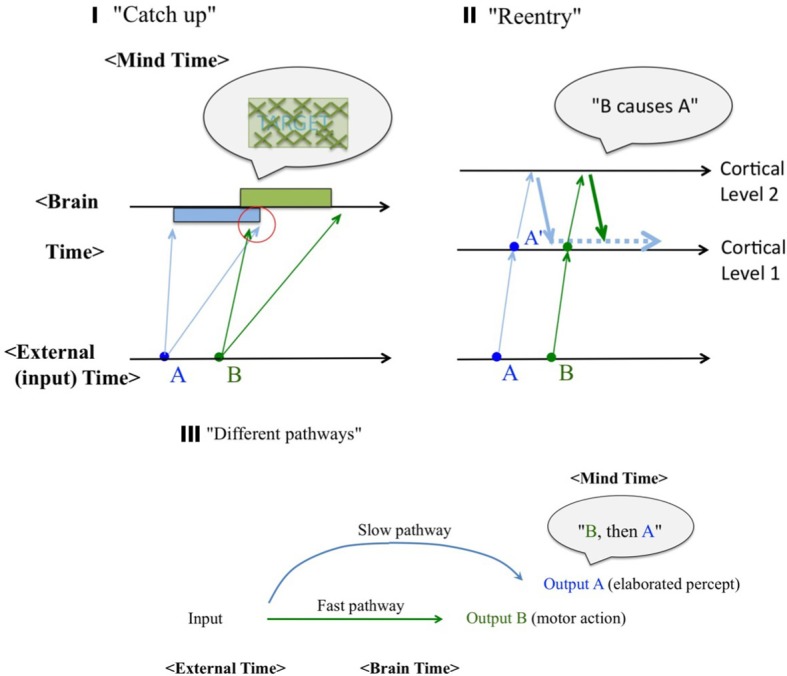
**Schematic diagrams of possible mechanisms.** External (environmental) Time, Brain (neural/physiological) Time, and Mind Time are represented separately. **(I)** The “catch up” model. Different speeds of neural signal conductance were expressed by fanning arrows, for each of the sensory events (A and B). Under certain conditions, fast neural signals coming from the physically subsequent event (B) may catch up slow neural signals from the prior event (A) to causally affect its perceptual consequence, as indicated in the red circle. **(II)** The “reentry” model. Feedforward signals from the cortical level 1 are sent back from the higher level 2 to the level 1, thus enables contextual effects from both surrounding and subsequent stimuli. **(III)** The “different pathways” model. The identical stimulus can generate two distinctive output responses A (an elaborated percept) and B (typically a motor action), each of which is mediated by two different pathways. Cortical pathways are typically (but not always) considered the “slow and conscious” whereas subcortical pathways are often considered the “fast and subconscious.”

We have reviewed backward phenomena using our own definition at sensory/perceptual levels. It may be the time to consider what alternative we have, in terms of possible neural mechanisms. Albeit schematic, we can list some, as illustrated in Figure 6. External (environmental, or physical stimulation) Time, Brain (neural/physiological) Time, and Mind Time are represented separately in these diagrams. The oblique arrows denote neural conductance delays (the more oblique from the vertical direction, the slower).

A remark may be necessary here, with regard to the distinction between the Brain Time and the Mind Time. “Mind Time” will be used as a short name for “mental representation of the temporal events.” Most of scientists naively assume that the Brain Time defines the Mental Time, and thus equate them, which the author cannot agree. A perceptual sequence of events, as a content of a percept, should be logically dissociated from the physical sequence of neural correlate events which caused them. When an event A is perceived prior to another event B (“A→B”), such a stream of percept (“A→B”) should also have a neural correlates. The neural correlates, however, does not have to be in the form such that there are two dissociable neural events corresponding to A and B respectively, nor that they are in this physical sequential order [the neural event(A) → neural event(B)], although such one-to-one mapping between the perceptual events and the neural events may be found at the peripheral or the lower-level visual representations. This point may not seem to be necessary in this section, but the significance will be clearer when we argue against the “first-order isomorphism” in the temporal domain and Benjamin Libet's view later (section Libet's Claims, and the “free will” Endangered? and “Sense of agency” as Postdictive Attribution and an Authentic Illusion).

The first intuitive option is the “catch up” model (Figure 6I). It has been accepted that the same retinal input may arrive at the primary visual cortex with various timings, and the same may be applied to the lower and upper levels of the visual hierarchy in general. Thus, a fast signal of a physically subsequent stimulus B may catch up with a slow signal of a stimulus A to affect the percept of it causally (e.g., the visibility of it, as in the case of backward masking; Breitmeyer and Williams, 1990), as indicated in the red circle in the figure. The slow and the fast signals have been associated with either X and Y channels, or sustained (P) and transient (M) channels (e.g., Breitmeyer, 1993) in terms of the neural implementation. It may appear confusing to some readers because this model solely based on feedforward pathways, yet claimed to be a potential account for postdiction. Note once again that throughout this paper, the definition of the postdictive phenomena is strictly operational (section Backward Perceptual Phenomena), and the proposed mechanism can be either feedforward like this, or re-entry (as the next model) which can be considered postdictive at the implementation level, or even more explicitly postdictive as the Benjamin Libet's model (as will be described in section Libet's Claims, and the “free will” Endangered?).

Figure 6II denotes an alternative idea (“reentry”), which assumes vigorous feedback from a higher level to a lower level of the visual information processing hierarchy. It is such feedback pathways that enable various sorts of contextual effects, including some postdictition (as indicated by the thick blue and green downward arrows) and even conscious awareness (Lamme, 2001; Fahren fort et al., 2007). This may allow more room to account for paradoxical causal perception, as will be described later (section Spatial Memory Updating with Perception). Both the “catch up” and the “reentry” models have been entertained especially for the backward masking and the flash lag.

The third option (“different pathways”) heavily relies on the known dichotomy of two visual pathways (ventral vs. dorsal, “what” vs. “where” or “cognition” vs. “action”; Goodale and Milner, 1992). This scheme is meant to explain dissociative, or selective deficiency in patients, as well as differences between explicit and implicit measures (such as reflexive reaction times vs. elaborated, conscious perception; e.g., Vorberg et al., 2003). However, it can also be applied to account for some of the seemingly paradoxical, postdictive phenomena, as will be described later (Neural and Computational Considerations). For a real-world example, competitive 100 m sprinters often report that their legs start moving even before their conscious awareness of the starter's pistol sound. It can be interpreted with multisensory prior entry, i.e., a difference in neural delay in different sensory-motor pathways, such as auditory→motor vs. motor→kinesthetic. If so, this actually reflect a rare failure in the ordinary postdictive reconstruction process of causality, as “the pistol sound triggered my leg reaction,” thus allowing us a glimpse into what is normally occurring a the implicit level, before the postdictive process operates (we will be back again to a similar real-world example in section Libet's Claims, and the “free will” Endangered? and Figure 10II). Given that this model incorporates global pathways/connectivity aspects of the brain, it may have more flexibility to account for paradoxical causality like this.

As the fourth option, we can add the “memory revision” model (Dennett and Kinsbourne, 1992), in which a tentatively established memory representation may be revised later. The object updating idea (described in section Flash-lag Effect, its Variations, and Object Updating) may be considered a specific example of it. This model may be more appropriate for the phenomena with a longer time scale, as will be described in the next and the subsequent sections.

These concepts exemplify the prototypical ideas of mechanisms underlying various sorts of postdictive phenomena. They are not necessarily mutually exclusive, especially because some tap into existing neural mechanisms while others emphasize more hypothetical, theoretical structures. More recent models may be considered as hybrids. For example, Bachmann's (2013)“perceptual retouch” model seems to have incorporated both the “reentry” and the “different pathway” ideas. Likewise, the fourth model, i.e., the revision of memory, may be involved more or less in all the other models (although it depends on the definition of “memory”) because those inevitably refer to some neural representation of sensory input, which may be called memory (albeit very iconic, or short-term). The distinction between perception and memory may be important when one discusses neural implementation, but it will be made less important when we will extend this review to a longer time scale because of the similarity and the continuity in function and abstract structure (section Extending the “postdiction” Concept to the Memory and the Sensory Consequences of Voluntary Movements and Hindsight Bias, and Cognitive Consistency).

What is also noteworthy here is that some of these models (especially the first and the third) are rather conservative, in that temporal sequence of the relevant neural events can directly determine and thus be “read out” as the perceived order (and in some cases causality) of the perceived event. Thus once again, “non-postdictive” (such as the “catch up” and the different “pathway”) models as well as “postdictive” neural implementations (such as the “re-entry”) can potentially offer alternative accounts for the “postdictive” phenomena in its operational definition.

For the rest of this paper, we will every now and then refer back to these diagrams. When we discuss the relevance of Benjamin Libet's claims, especially the “backward referral” claim, we will point out some potential problems related to “the first-order isomorphism” between the Brain and Mind Times, that is implicitly assumed particularly at microscopic time scales in these models (with the possible exception of the memory revision model). A more intriguing possibility based on a strict distinction between perceived timing as a content of perception vs. its physical timing of its neural correlates, will be introduced.

Thus far, we have discussed about vigorous postdictive reorganization in the time scale of hundreds of milliseconds, whereas now we will include memory updating and perceptual reorganization on a time scale of one to several seconds (section Extending the “postdiction” Concept to the Memory and the Sensory Consequences of Voluntary Movements), as well as higher cognitive functions including hindsight in visual exploration/detection, and the postdictive reconstruction of causal attribution in long-term memory, where the relevant time scale will range from minutes to days (section Hindsight Bias, and Cognitive Consistency).

The extension of our list of postdictive phenomena into the longer-time scale, and memory will have two implications. First, it will point to the possibility that the postdiction may be a very general principle from sensation to cognition to memory, and with time delays from tens of millisecond to months of time delay (Neural and Computational Considerations). Second, it will make it more feasible to consider visual awareness as extra-short (iconic) visual memory, which is phenomenologically and structurally continuous to short-term memory. For an intuitive example, consider a “percept” of flickering light. It is directly “perceived” as such, but some form of memory is logically necessary “to perceive” it.

## Extending the “postdiction” concept to the memory and the sensory consequences of voluntary movements

Perceptual events are constantly consolidated into memory, but the transition process is not precisely akin to simply creating a Xerox copy. Instead of faithfully duplicating the perceptual structure at the time, it rather reorganizes the event sequence in accordance with various principles, such as information compression, better Gestalt, consistency with regard to the relevant context, and a causal framework, etc. Wu et al. (2009), for example, demonstrated that a flash that actually caused reappearance of the target stimulus in awareness (after having been “subliminated” by motion induced blindness, Bonneh et al., 2002) was itself consciously perceived as appearing later than the reappearing target. Thus in this case (as many other cases dealt with in the current paper), perceived temporal sequence of the two events are detached and inconsistent with the physical causality. Note that the “catch up” model (in section Underlying Neural Mechanisms?) may suffice to explain the illusory temporal order, but a conscious percept may require more, including causal attribution at least in some cases. Wu et al. (2009) prefer the reentry model to explain, but there may be another account feasible based on a neural delay difference and a distinction of specific/nonspecific processes (Bachmann and Aru, 2009).

This type of backward cognitive reorganization has been reported repeatedly in cognitive and social psychology. For example, F. C. Bartlett in his classical study (1932) used American Indians' folktales as materials to recall, which may appear illogical or unrealistic to average Westerners, in a recall experiment. Recalled stories by British participants (students) revealed some distinctive eliminations, re-ordering, and biases to make the stories more consistent and logical. In their seminal series of studies, E. F. Loftus and her group (1979) demonstrated that witnesses' memories of an accident can be biased by the way of questions/instructions and by the context and episodic memory of recall itself. Memory was reorganized mainly for consistency, information compression, and ease to of retrieval in these cases. In some cases it can be interpreted just as a simple confusion on temporal sequence, but in most cases, the causal interpretation or even a revision of the content of memory is involved. Similar causal misattribution/memory modification has been observed when one is asked for “intention” of action as the cause of a movement (as will be mentioned again in section “Sense of agency” as Postdictive Attribution and an Authentic Illusion).

Memory reorganization of this type is commonly known, but the current question is as follows—Could the same type of postdictive reconstruction of memory occur at a much lower sensory-motor level, and in a much shorter time scale? If such a case exists, then it would bridge the gap between the backward perceptual phenomena (reviewed in the previous sections) and memory, raising the intriguing theoretical possibility that the postdictive construction be a general neural-cognitive principle that governs from lower sensory to higher cognitive processes, from micro to macroscopic time scales.

### Spatial memory updating with perception

**Figure 7:**
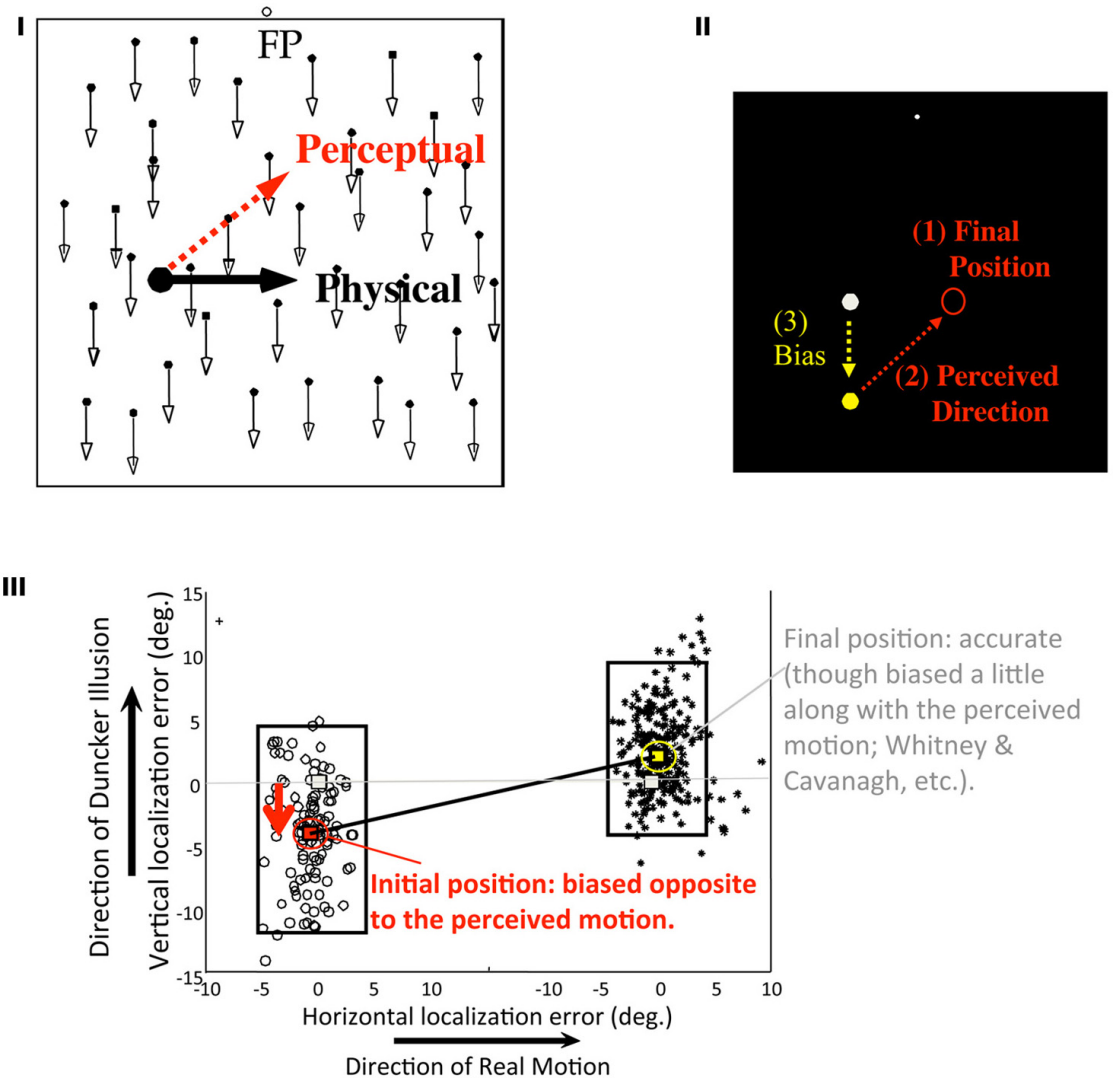
**Memory updating with perception (Sheth and Shimojo, 2000). (I)** Stimulus configuration. While the participant maintained eye fixation, the target moves horizontally. Due to the background dot pattern movement (either upwards or downwards), it appeared to move in an oblique direction (called “Duncker illusion,” or induced motion). Upon the stimulus offset, the participant localized either the target's initial or the final positions (depending on a tone cue given at that moment) by pointing and clicking with a mouse cursor. **(II)** Response (the initial position estimation) expected in the case of a full postdictive reconstruction. Since the participant was more certain about the exact location of the final position of the target, and also since the oblique trajectory of movement due to the illusion was so compelling, we hypothesized that the participant would bias the memorized position of the target at onset, in the direction consistent with the illusory trajectory (as shown in the diagram). **(III)** As the results, vertical and horizontal localization errors were plotted. Each dot represents a single trial. The length of the rectangle indicates the standard error of the initial and the final localizations, respectively. As can be seen from the figure, the final position was deviated relatively little (right), but the initial position was biased opposite to the illusory bias of the motion trajectory, as expected (left). The differences in localization error between the initial and the final positions were highly significant, in terms of both accuracy (*P* < 10^−7^) and directional bias (*p* < 10^−30^; *N* = 7). (Figures are modified from Sheth and Shimojo, 2000, Figures 1 and 2.)

In Figure 7I (revised from Sheth and Shimojo, 2000), a target dot undergoes a smooth translational motion at a constant speed from the left to the right on a CRT display. When it disappears, a tone plays with either a high or a low pitch randomly. Depending on the tone pitch, the participant in the experiment was asked to report either the initial, or the final position of the target respectively, by moving the cursor and clicking the button as soon as possible. The stimuli and the task were as simple as such, except for one critical aspect that is, a random dot texture, which moved either downwards or upwards randomly, was added to the display. Due to the well-known “Duncker illusion,” a target that physically moved horizontally appeared to move obliquely upwards (the red arrow in Figure 7I; against the background dots moving downwards) or obliquely downwards (against the background upwards). Would the memorized initial position be affected by this illusory bias of the trajectory? More specifically, would the bias of the initial location be in a direction that was more consistent with (1) the final position which should be the latest and thus more accurate visual signal, and (2) the perceived (illusory) direction of the trajectory (as illustrated in Figure 7II)?—These were the critical questions that we raised with this paradigm.

Figure 7III shows the results Sheth and Shimojo (2000). As expected, the errors in the final positions were relatively small (right), but the initial positions (left) were biased significantly in the direction consistent with that of the perceived trajectory and the final position. Because the participants had learned quickly via the practice and in the initial trials that they would be asked for the initial position with a 50% probability, it can be assumed to be a trivial cognitive strategy for them simply to memorize the initial position as accurately as they could at the outset in the each trial. In the result, the bias was substantially smaller than what expected from a complete compensation to be consistent with the perceived trajectory, but it was significantly above zero.

Several control experiments revealed further that: (a) making known the nature of the illusion, or (b) making the trajectory of target motion much more irregular and complicated (to minimize a straightforward, conscious and logical inverse calculation), did not significantly reduce the bias. Moreover, (c) reducing the latency of the response (i.e., allowing the subject to respond immediately when they saw the beginning of the target motion) reduced the bias substantially, but not completely.

This type of spatial memory updating has two significant implications, at least. First, as emphasized previously, it indicates a constant updating process of memory when faced with real-time sensory inputs. Second, it may indicate the “revising” of causal perception, albeit implicitly. That is, the initial location, the trajectory, and the final location are reorganized in a more consistent causal framework of perception in this case. Thus, it may share implications with several other studies concerning causality perception. For examples, on top of Wu et al. (2009) that is described in section Extending the “postdiction” Concept to the Memory and the Sensory Consequences of Voluntary Movements, Choi and Scholl (2006) demonstrated that visual events can determine whether a collision is perceived in an ambiguous situation even when those events occur after the moment of “impact” of the putative collision has already passed. Thus, the findings overall indicate a vigorous automatic tendency of updating short-term memory to be consistent with on-line perceptual inputs, even at this simplest and lowest sensory level. This immediately raises a related question as to whether this type of postdictive reconstruction occurs only in positional information, or whether it may occur in any other visual attributes, such as shape or color? The logical expectation, especially from the “generalized flash lag” observation (section TMS-induced Scotoma, and Backward Filling-in), would be the latter because there is nothing intrinsically special about position in this case (i.e., dynamic reconstruction). Albeit inconclusive, we do have some evidence consistent with this expectation as described in the next section.

### Pursuit mislocalization, and effects of the spatial context

**Figure 8:**
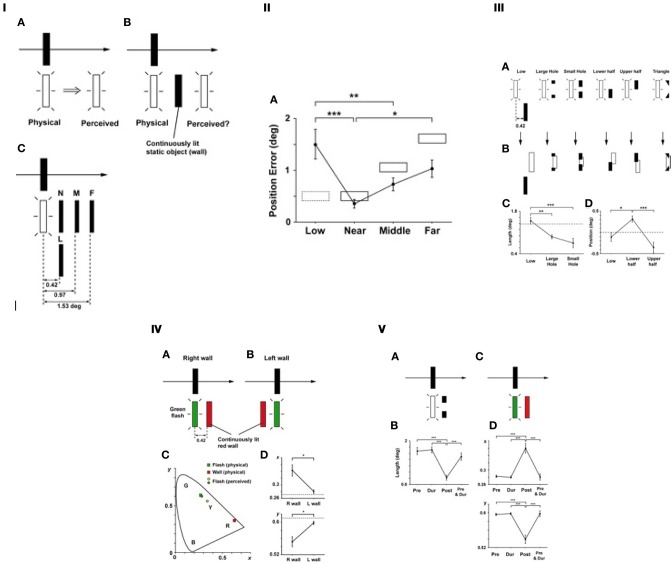
**Saccade mislocalization, and effects of spatial context (adopted from Noguchi et al., 2007). (I)** The main experiment. **(A)** The basic effect of the pursuit mislocalization. The black arrow indicates the direction of both the target's (black) movement and smooth pursuit eye movement, while the white arrow indicates the mislocalization effect. **(B)** The main experimental configuration where a static “obstacle” was present in the trajectory of mislocalization throughout the trial. **(C)** Four different locations of the obstacle, as the main variable. **(II)** Results. Position errors (deg.) are plotted for the spatial conditions of the obstacle. The solid and dotted rectangles indicate the location of the wall in each condition. As can be seen, the position errors were the largest in the low condition (with no significant reduction), then smaller in the far, the middle, and the near conditions in this order. This was exactly what should be expected from the topographical “spatial representation” idea. **(III)** Manipulations to partially occlude the trajectory zone **(A)**, phenomenological results **(B)**, and more quantitative results plotted as length **(C)** and position **(D)** of the perceived target. **(IV)** Color mixture. The two stimulus configurations/ sequences employed **(A,B)**, and the results in the CIE xy color space **(C,D)** were shown. As can be seen in **(C)**, the colors were mixed into a subjective yellow. As can be seen in **(D)**, the color mixture effect was much larger in the “right wall” condition (**A**, where the obstacle was located right in the middle of the trajectory) than the “left wall” (**B**, where the obstacle was behind it). **(V)** Effect of timing. We compared four different timing conditions: (a) Pre, (b) During (the flash target presentation), (c) Post (during + after), and (d) Pre + Dur. In the partial occlusion (“a hole”) variation **(A)**, the effect of blocking the mislocalization was largest in the Post condition **(B)**. In the color mixture variation **(C)**, the mixture effect was maximum also in the Post condition **(D,E)**. (Reproduced from Noguchi et al., 2007, Figures 1, 2, 3, 6 and 7, with permission from ARVO.)

Pursuit eye movement on a smoothly moving object leads to a mislocalization of the target that is briefly presented nearby during the pursuit (Mitrani and Dimitrov, 1982). To be more specific, the direction of mislocalization is in the direction of the pursuit movement (Figure 8IA). What if there is an obstacle (a continuously present static object) in the trajectory of the mislocalization (Figure 8IB)? It would be inconsistent if the brain has a spatial representation in which it has to carry the location of the flashed target along the translational trajectory. How would the brain resolve such an inconsistent situation? This was the motivation of the experiments (Noguchi et al., 2007). Directly extending the implications of the previous study (with the Duncker illusion, in the previous section), one may hypothesize that the visual system pursues a more consistent interpretation of spatio-temporal events, modifying the natural tendency of the pursuit-caused spatial bias. Figure 8IC illustrates one variable, which is the position of an obstacle relative to that of a flashed target, and Figure 8IIA shows the results where the positional errors are plotted against the relative positions. Essentially, the mislocalization was “stopped” by the obstacle, but only if it is within the trajectory of the mislocalization. Likewise, when the obstacle was indeed in the trajectory of the mislocalization but only partially covering the length of the flashed target (Figure 8III), the perceived mislocalization is consistent with it in terms of the shape and the position of the mislocalized target in the spatial representation (Figure 8III).

More intriguing was when the obstacle had a different color (e.g., red) from that of the flashed target (green). As shown in Figure 8IV, a color mixture resulted. Note that while the color perceived was a mixture, that the mixed hue itself was never presented to the retina, which should be considered very convincing evidence for integration of signals within a temporal window. Note that the differently-colored obstacle needed to be located in the direction of the mislocalization (Figure 8IVA), not elsewhere (B). This effectively eliminates the possibility of any local aftereffect.

A related observation was made in the flash lag circumstance, where a red target was flashed exactly on top of a green object, for instance. This would yield an yellow percept due to color mixture normally, but when the green object underwent a smooth motion (either rotational or translational), the red flash was mislocalized and at the same time seen qualitatively very close to the original saturated red (Nijhawan, 1997). Therefore, in this case, color decomposition instead of color mixture (of the retinal inputs) occurred. What is common between these two cases, the smooth pursuit mislocalization and the flash lag, is that the color perceived was seemingly consistent with the perceptual localization, as opposed to the retinal.

In the study of pursuit-driven mislocalization, we also manipulated the timing of the obstacle with regard to that of the target. The results (Figure 8V) suggested that the reorganization of the shape and the color were maximal when the obstacle was presented in the “post” period (i.e., having the same onset as the flash, but lasting longer after the flash offset), relative to the “pre,” “during” or “pre and during” periods. This suggests that the “carrying over” mechanism for the localization of the flashed target operates beyond the duration of the flashed target itself, and that the presence of the obstacle interferes with it in the critical time zone. Nonetheless, the resulting consistent features (i.e., positions, shapes and colors) are perceptually “backward-referred” to the moment of the flashed target—backward-referred because it is phenomenologically not the case that the original positions/colors/shapes are perceived first, and then re-perceived as modified. Rather, all of those “reconstructed” features are perceptually given as a one-shot, immediate percept from its onset of appearance. The similarity to the flash lag (especially the “flash initiated” case) should be obvious.

Thus, the postdictive reconstruction occurs in not only the position, but rather in various visual attributes including, the shape and color (and even the temporal order). Together with the generalized flash lag effect (section Flash-lag Effect, its Variations, and Object Updating) and the memory updating results with the Dunker illusion (section Spatial Memory Updating with Perception), in terms of postdictive processing the position is not special. Rather, all the concurrent visual feature information is dynamically and iteratively processed to reach a consistent scene interpretation at the given moment.

## Neural and computational considerations

Here, we would like to reconsider the possible mechanisms (section Underlying Neural Mechanisms?), but this time with more explicit references to specific neural mechanisms, with additional computational thoughts especially in the extended time range.

As mentioned in section Underlying Neural Mechanisms?, varying speeds of neural signal conductance even within the same pathway (say, from the retina to the V1 via the LGN) are well established (e.g., “Parvo” vs. “Magno” pathways; Livingstone and Hubel, 1988), providing the basis of the “catch up” idea (Figure 6I).

More recent advances in neuroscience implicate reentrant signaling as the predominant form of communication between brain areas, and this idea may help us to understand the neural correlates of visual awareness, in situations such as backward masking (Di Lillo et al., 2000). To be more specific, they identified two masking processes both of which are based on reentrant signaling. One is an early process that is affected by physical factors such as adapting to luminance, and the other is a later process that is the masking by object substitution. Iterative reentrant processing, when formalized in a computational model, provides a more comprehensible account of all forms of visual masking than do the long-held feedforward views based on inhibitory contour interactions. Along this line, V. Lamme and his colleagues revealed that the EEG derivatives that are typically associated with reentrant processing were absent in the masked, as opposed to non-masked, condition (Fahrentfort et al., 2007; although there is a notable objection, e.g., Põder et al., 2013). A study employing TMS with the metacontrast paradigm suggests that a prior visual stimulus can influence subsequent perception at the early stages of visual encoding via feedback projections (Ro et al., 2003). In the context of “blind sight,” there is substantial evidence in favor of the theory that unconscious visuo-motor transformations, as in the blindsight, are executed in an entirely feedforward processing cycle, while visual awareness is critically dependent on feedback connections to the primary visual cortex (Lamme, 2001).

These findings make reentrant signaling as a good candidate for the postdictive phenomena described thus far in this paper (Figure 6II), for a variety of reasons. First, the reentrant feedback is appealing intuitively in the sense that an earlier (in both the temporal sense and the visual information processing hierarchy) visual representation is “revised” by the feedback from a higher level. Second, the distinction between the implicit vs. the explicit processes may nicely map onto the feedforward/feedback distinction (as shown in the case of blind sight above). Last but not the least, such reentrant signaling may in principle occur from very short-ranges (such as different layers of the visual cortex, or neighboring visual areas such as V1 and V2) to very long-ranges (such as occipito-frontal and occipito-temporal connections). This last point is especially significant in the current paper, which aims to find a common thread in various postdictive phenomena, across very different temporal and neural scales.

Finally, the idea of two major, dissociable visual streams has been presented. Whereas Mishkin et al. (1983) characterized the ventral and the dorsal pathways “what” vs. “where,” Goodale and Milner later modified as “cognition” vs. action with new patient data. This provided the basis for the “different pathways” idea (Figure 6III).

From a more computational viewpoint, at least some of the postdictive phenomena may be understood in the Bayesian framework, where the conditional probability indicates signal-to-noise ratio in the visual input while the prior probability may be encoded in the prior internal state of the relevant brain region. Indeed, a similar attempt to account for the rabbit and some other postdictive effects in the Bayesian framework has been made elegantly (Goldreich and Tong, 2013). It is also consistent with the general implications from the TMS studies (reviewed in section TMS-induced Scotoma, and Backward Filling-in) in which a conscious percept reflects both the retinal input (as a likelihood) and the internal neural state (as a prior). More specifically, some additional (potentially arbitrary) assumptions may be necessary to be consistent with the findings. The occurrance of the scotoma itself is due to a local disruption of topographic representation of the retinal input (i.e., a local blockage of the likelihood). There is evidence that the TMS locally suppresses the retinotopic mapping of the visual field on the surface of the visual cortex (Kamitani and Shimojo, 1999) so this assumption is reasonable. Then, the backward filling-in may simply reflect the brain's tendency to rely heavily on the prior (whichever information internally available at the critical moment) when the likelihood is locally not available or very noisy. The Bayesian may provide an overarching framework to more explicitly formalize the postdictive phenomena across the wide range of time scale (from sensation, to perception to cognition).

The idea concerning “compensation of a neural delay by extrapolation” in the flash lag (Nijhawan, 1994) may also be considered in this framework, where expectation or prediction (or a “set” in a higher cognitive term) is implemented in the internal state (as suggested in Berkes et al. (2011) and de Lange et al. (2013), for example).

As for the big picture, more complex brains have more reentrant connections, thus enabling Bayesian-like complex decisions, postdictive reconstructions, and possibly “awareness.”

## Hindsight bias, and cognitive consistency

As mentioned above, there is a rich source of evidence of cognitive reorganization for consistency, information compression, and ease of recall. In the social science literature, a similar effect is known as “hindsight bias.” Hindsight bias is the tendency to retrospectively think of outcomes as being more foreseeable than they actually were. It is a robust judgment bias and is difficult to correct (or “debias”). It has been demonstrated in historical events as well. For example, people retrospectively overestimated how well they could predict the restoration of US-China relations during the period of Nixon's surprise visit to China, as opposed to their actual predictions during the visit. So this is a cognitive postdiction phenomena in a large time scale, where people tend to implicitly “revise” their memory on prediction in the past under the influence of the outcome. (The study on athelete's “sixth sense” which will be described in the next sub-section is also in the same format).

**Figure 9:**
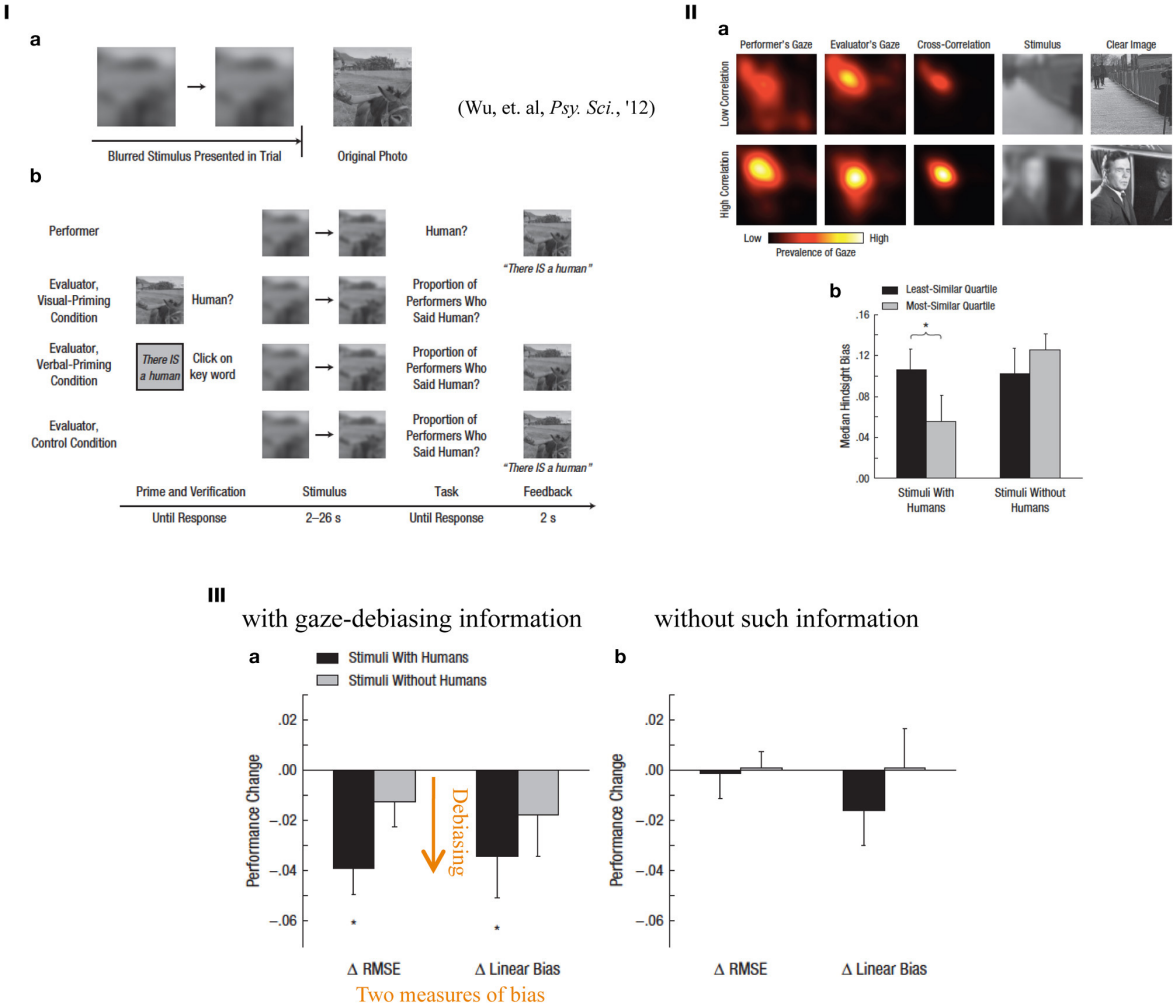
**(I)** Hindsight; procedures. The task instruction was the following: “An image was getting sharper. Decide if there is a human ASAP.” The performers just had to do this task, whereas the evaluators had to evaluate “proportion of performers said human (present)” with a visual, or a verbal priming. **(II)** A comparison of eye movement patterns and task performance. (a) The top raw shows an example of “low correlation” between the performer's and the evaluator's gaze patterns as heat maps, with the stimulus and original (clear) photo image. The bottom raw shows an example of “high correlation” stimulus. (b) Median hindsight biases plotted for each conditions (stimuli with/without humans). Black bars show the result of least similar (lowest correlation) quartile, while gray bars show that of most similar (highest correlation). “^*^” Represents statistical significance between xxx (*p* < xx). **(III)** How much debiasing effects were obtained are shown either with (left) or without (right) gaze pattern information of the performers. Two different quantitative measures (ΔRMSE and Δlinear bias) of bias produced similar results. Black bars denote stimuli with humans, whereas gray bars denote stimuli without. “^*^” Represents statistical significance from zero (*p* < 0.05) (reproduced from Wu et al., 2012, Figures 1, 3 and 4, with permission from Assoc. Psychol Sci.)

Hindsight bias may explain the cognitive gap between those who are accused vs. those who accuse them in a medical law suit or after a more large-scale disaster such as a nuclear plant accident, because the accusers accuse the accuses always based on their retrospective, thus postdictive, estimation of how much prediction was possible on the disastrous outcome, only after it occurred. The author and his colleagues became interested in a situation in which one who was informed regarding a problem situation tended to overestimate how much an uninformed could perform a perceptual task. In the experiments, we used a visual paradigm in which performers decided whether blurred photos contained humans, while the image was progressively made sharper (Figure 9I; Wu et al., 2012). Evaluators, who saw the photos unblurred (visually primed) or verbally primed thus knew the correct answer (a human present/absent), estimated the proportion of participants who would guess whether a human was present at a given degree of defocus. The evaluators exhibited visual hindsight bias, i.e., an overestimation of judgment performance by the uninformed participants (the data not shown; Wu et al., 2012), but only with a visual priming, not with a verbal priming. It can be again considered a form of cognitive postdictive bias because the known answer (presence or absence of a human) substantially affects the estimation of the task difficulty *before* knowing the correct answer (although in this case the estimation was on some others' performance, not the informed own). The data qualitatively and structurally matched earlier data on judgments of historical events surprisingly closely. Using eye tracking, we further showed that a higher correlation between the gaze patterns of performers and evaluators (shared attention; as indicated in the heat map in Figure 9IIa) is associated with lower hindsight bias in the stimuli with humans (Figure 9IIb). This association was validated by a causal method for debiasing: showing the gaze patterns of the performers to the evaluators as they viewed the stimuli progressively reduced the extent of hindsight bias, as indicated in two different measures of performance change (Figure 9III).

The study suggests that task difficulty/performance is often re-constructed retrospectively. The exact neural mechanism underlying such long-term cognitive hindsight bias would be different from that which underlies perceptual backward phenomena on the microscopic time scale. Nonetheless, the similarity in the results between these types of visual and the historical tasks indicate, at a functional level, that they may reflect a general intrinsic tendency of the brain to learn from experiences but exclusively in the cognitive format of “cause and effect” such that it can be used for adaptive predictions in the future.

### An athlete's “sixth sense”?

For a further investigation of this type of postdiction, i.e., the re-construction of events into a cause-effect format in a more controlled way, and how such an automatic tendency overcomes the natural tendency to be consistent with one's own past decisions, we decided to examine athletes' “sixth sense” as to how well they predict they would do in the next game/match. Athletes in various sports, including top professionals and amateurs, often claim that they can tell whether they will be a hero or not in the next game/match, but is that a real prediction based on some implicit self-assessment of one's physiological and mental conditions, or simply a postdictive construction (which can occur when the question is raised only after the game or match)? We asked over 100 college and high school athletes in a variety of sports [volleyball, soccer, basketball, and Kendo (Japanese fencing)] to fill out a questionnaire in the morning before an actual game or match later in the day (Kadota et al., 2009). The question of our interest was embedded in other ordinary questions about their mental and physical conditions, their teamwork, etc., asking “How do you think you will perform today?” (Prediction). We then repeated a similar set of questions, including another question of our interest, “How did you think you would perform this morning?” (Postdictively-reconstructed prediction).

Virtually the same question was repeated within the same day within subjects, thus it should have been easy for the participants to notice their own inconsistency. Nonetheless, more than a half of the athletes who participated changed their prediction in the postdictively reconstructed case. Moreover, those who lost tended to make their changed predictions more negative, whereas those who won tended to make them more positive. The tendency of interaction was highly significant (*p* < 0.005). On the other hand, neither the predictions before the game, nor answers to other questions (such as mental and physical conditions), nor physiological measures (such as body temperature, hear rate, and blood pressure) did accurately predicted the performance, according to a path analysis performed later. The overall pattern of the results went against the natural tendency to be consistent when answering the postdictive question with the memory of the predictive, strongly indicating a robust tendency at an automatic and implicit level, of postdictive reconstruction to be consistent with the actual outcome. Such automatic and implicit characteristics thus hold generally, from the sensory to the more cognitive levels.

### Real-world implications

These studies described above have obvious social-scientific implications because the hindsight bias can be a cause of various sorts of conflict in employer-employee relationship, sports, medial lawsuits, and even international affairs. It may even cast a doubt on some “scientific” studies in other fields. For example, millions of dollars of federal science budget have been spent in China, Japan and various European countries to explore the possibility of “predicting” massive earthquakes from certain “signs.” An intuitive part of the motivation behind this came from anecdotal reports of observations such as abnormal animal behavior or natural phenomena (such as unusual shapes of clouds or an extra bright sunset, etc.) as a possible precursor to the disaster. The fundamental problem with these reports, needless to say, is that those episodes were collected only after the earthquake with no exception, making them highly susceptible to postdictive biases. Formalistically, the conditional probability of such a large earthquake to occur, given such an “unnatural” sign reported in a *post-hoc* fashion, should be compared with a conditional probability calculated via daily (prior) observations; that is, given a pre-designated unnatural sign in one morning, what was the chance that a major earthquake would occur later on that day (or a predefined short time period). The latter type of data would be very difficult practically to collect (because it would require enormous amount of time and resources), and perhaps may never exist.

What did we learn thus far in this review? First, there are various cases in the perceptual domain in which a conscious percept is based on some integration process in a limited temporal time window (of approximately 100–200 ms), within which a stimulus presented later can seemingly affect causally how the subsequent stimulus is perceived. Second, conscious perception can thus be equated to a sort of “ultra-short-term” (iconic) memory, except that against the classical concept of a passive, faithfully duplicated but fainting copy of the original input, this process should be considered to be a very dynamic reconstruction from a sequence of sensory inputs. Third, there are several prototypical mechanisms conceivable, such as the “catch up,” the “reentry,” the “different pathway,” and the “memory revision” models, each of which has reasonable behavioral/neural evidence behind it. Fourth, the structurally similar postdictive reconstruction seems to occur as well on a much larger temporal scale, in the domain of retrospective causality attribution and the postdictive reconstruction of a prediction, which may characterize complex brains.

Regarding the last remark, we have used the term “reconstruction” repeatedly, but it is not meant to imply the repeated experience of the conscious percept itself. Instead, the reconstruction process may be postulated in the following way. In the first implicit stage, there may be a faithful representation of a physical event sequence at earlier implicit levels of information processing. It is only at the later levels, a downstream of the information processing or along a different pathway, where a conscious percept is constructed (for the first time) such that it is more consistent with a context including the subsequent stimuli and a causal framework of cognition.

This last point should be taken seriously, as it implies both the presence of an implicit, automatic predictive process, as well as a reconstructive, postdictive process for conscious perception.

## Libet's claims, and the “free will” endangered?

Benjamin Libet made several important observations and claims which are highly relevant to the central thesis of the current paper, i.e., postdiction (Libet, 2004).

The first of these involves his simple observations with a train of electric pulses to stimulate the somatosensory cortex of the human patients. He observed that a sensation generated by a weak electric pulse (just above the threshold) can be suppressed “backwards” by a train of pulses applied with a 200–500 ms delay. If the initial stimulus is repeated within a several-second interval however, a cutaneous sensation is rather facilitated by the same subsequent train pulses with the same 200–500 ms delay. The relevance of the observations is obvious because these are considered to be another example of postdiction, but this is more related to the TMS example above (section TMS-induced Scotoma, and Backward Filling-in) because in both cases, a direct neural intervention causally affects the percept of a stimulus presented earlier (although the former case is in vision, while this is in the cutaneous modality).

**Figure 10:**
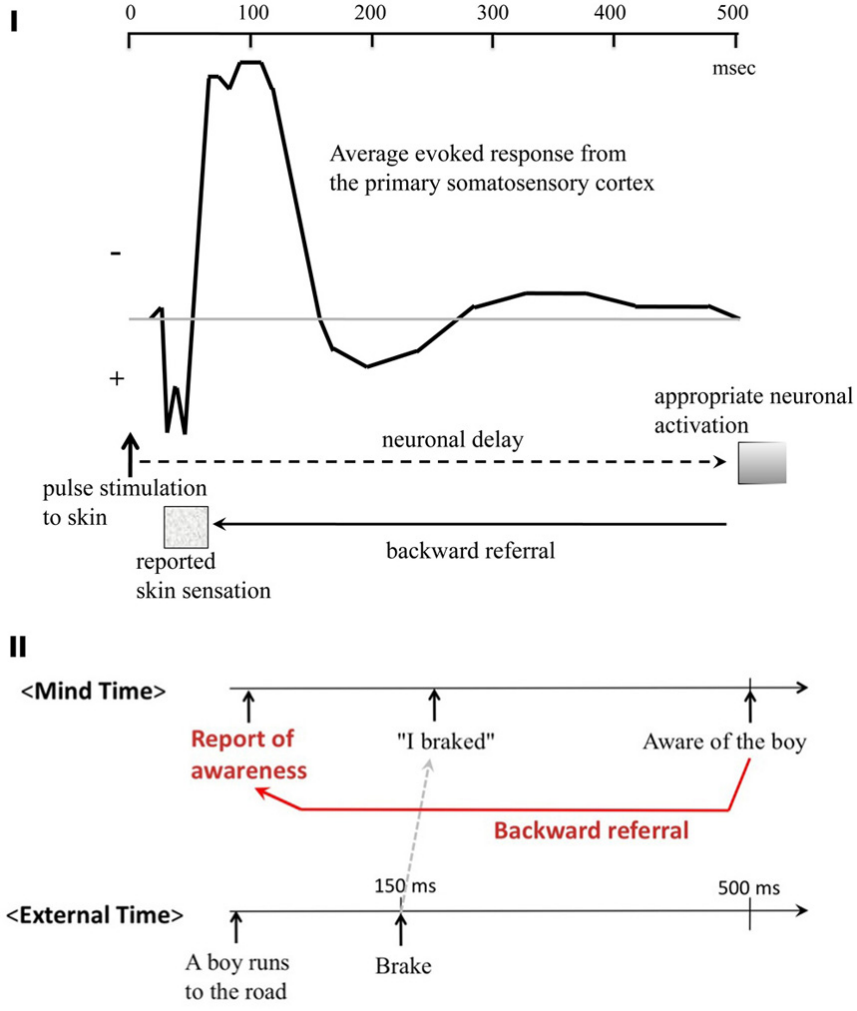
**Benjamin Libet's findings on postdictive process, and backward referral. (I)** Time marker for the backward referral. The first peak of the evoked electric response from the primary somatosensory cortex is quick, temporarily locked to the stimulus onset, and present even when the stimulus is below the sensory threshold. Thus, it could be a good candidate of the time marker, to which the backward referral of the sensation caused by the sustained cortical activity occur. (Modified from Libet, 2004.) **(II)** Libet's functional account of the backward referral in the real world. The figure illustrates time sequences of external and mental events in 500 ms or so. When a driver hit the break because (s)he saw a small boy running into the road ahead of his/her car, his/her conscious report of the event sequence would be exactly in this order (as illustrated in red in the top raw). However, what actually happened with regard to the implicit and explicit levels of his/her mind would be different. It was rather likely that his implicit sensory-motor pathway had triggered the brake immediately (within 150 ms or so; as indicated by the gray dashed arrow), even before he was consciously aware of the presence and the content of the sudden object, i.e. the boy (as indicated at the top right). According to Libet, this scenario is well supported by a variety of laboratory evidence indicating presence of implicit and fast sensory-motor pathways. Thus, the backward referral process put the sequences of events into concise, cognitive frameworks such as causality and “intention of action.” (Modified from Libet, 2004.)

Second, in the same setup with direct current stimulation, he claimed that some implicit neural process precedes conscious perception, yet the onset of the conscious percept is perceptually “referred backwards” to the stimulus onset. He also pointed out that the first peak of the evoked potential recorded from the somatosensory cortex is a good candidate for the time marker, to which the backward referral occurs (Figure 10I).

Along this line, Nishida and Johnston (2002) recently re-examined Moutoussis and Zeki's observation (1997) of the asynchrony of color and motion percepts, arguing that the perceived timing of a sensory event should be strictly distinguished from the objective, physical timing of its neural correlates. To be more specific, they argue that even if the critical neural process of a visual attribute (say, color) is faster than another attribute (motion), it does not necessarily require that the former (color) appears earlier than the latter (motion) in the perceived sequential order. It is because the perceived sequence is the content of the percept in the Mind Time (in Figure 6), whereas the neural event sequence is in the Brain time. This critical distinction logically allows a room for Libet-type backward referral, and resolves its seemingly paradoxical (or even “anti-scientific” to some) appearance. By the same token, it effectively eliminates a “homunculus,” a mysterious Brain-Mind enigma who is sitting at the “brain center” to judge whether event A (color) or B (motion) occurs first. The same may apply to other postdictive phenomena, especially in the sensory-perceptual domain within 100 or 200 ms.

Libet's third claim concerns action. His findings on “preparatory potentials” suggest that there is specific neural activity that precedes and causally determines the execution of an action, in the order of several hundreds of millisecond. He also developed his own unique psychophysical paradigm in which a participant evaluated the timing of the onset of a conscious intention toward an action preceding its execution. Overall, he argued that the neural activity precedes and causes both the intention and the execution of action.

Why do we need such a complex process as backward referral? Moreover, how could we integrate his three claims into a general framework? Libet offers a functional account. He uses a real world example. The Figure 10II illustrates time sequences of external and mental events occurring within approximately 500 ms or so. When a driver hits the breaks because he sees a small boy running into the road ahead of his/her car, his conscious report of the event sequence would be precisely in this order (as labeled in red). However, what actually happened with regard to the implicit and explicit levels of his/her mind would be different. It was rather likely that his implicit sensory-motor pathway had triggered the brake immediately, even before he was consciously aware of the sudden appearance of the boy (as indicated at the bottom as External time line). This is akin to the other real-world example that we used earlier (section Underlying Neural Mechanisms?) of the 100 m sprinters who occasionally report their starting movements even before their conscious awareness of the sound.

According to Libet, this scenario is well supported by a variety of evidence indicating the presence of implicit and fast sensory-motor pathways. Thus, the backward referral process puts the sequences of events into concise, cognitive frameworks such as causality and “intention of action.”

Libet's claims generally injected some controversy into theories of the philosophy of mind and neuro-philosophy because it may (or not) endanger what is termed “free will,” which is to some the critical basis of legal responsibility in a democratic social system. Apparently, Libet himself suffered from this problem to put substantial efforts to rescue “freedom” from the implications of his own findings, relying on a concept of “vetoeing” of own intention (Libet, 2004), but it did not seem to be very successful. A different insight to resolve this difficulty actually comes by integrating his claims above, i.e., the implicit neural correlates preceding a conscious percept, and the backward referral of its perceived timing. Note especially that the backward referral may be considered an implicit, automatic (stimulus-driven) process of causal attribution. (Thus, causal attribution may not be always a higher level, cognitive process).

Finally, a remark on terminology may be necessary here to avoid a confusion. Throughout the current article, the term “postdictive phenomenon” is used strictly in the operational sense, as repeatedly mentioned above. However, the term “postdiction” sometimes refers to the “reentry” model or the Libet-type backward referral as the underlying mechanisms. One may want to make a clear distinction between these two usages.

The next section will be devoted to expounding the details of this idea of the (generalized) backward referral. Although it may seem to substantially exceed the specific scope of this paper, the author feels that this is necessary for a full understanding of the broad impacts of the findings discussed here.

## “sense of agency” as postdictive attribution and an authentic illusion

Based on the review and the discussion thus far, we have at least three lines of reasoning with which to believe in the compatibility of neuroscientific determinism and the spontaneity/volition of the human action. We will now examine them one after one.

1. The feeling of free choice may live in the postdictive process, not in the predictive process.
  The overwhelming majority of studies on perception, choice decision making and action have focused on the neural mechanisms that precedes and causally determines an action. However, there is a good possibility that psychological/neural processes after the decision may significantly contribute to determining whether a completed decision is felt as forced or more spontaneous/voluntary. Cognitive dissonance (Festinger, 1957), causal attribution (Heider, 1958), and choice justification (Staw, 1976) are some of the keywords in the social psychology literature which are potentially related. To state this simply, the sense of agency (or a feeling of free choice in a given situation) may well play out as a postdictive construct. This may be structurally similarly to the case of conscious perception, as in that case as well, a percept can be confirmed as “conscious” only when it is consolidated and reviewed (typically in a response to a question on the event). The challenging task for neuroscientists to account for the neural mechanism underlying the feeling of agency and “freedom” (and likely visual awareness as well) may not be accomplished until they shift their attention from the predictive process to the postdictive process.
2. *The feeling of free choice is a matter of content in perception/cognition. It should be distinguished strictly from the deterministic nature of the neural correlates.* For example, a content of “red” color perception is possible even though the neurons or the neuronal activity underlying that perception itself is in no physical sense colored red. When a part of somatosensory cortex is activated, the pain is not felt there, but rather felt at the “referred” body part. Likewise, the feeling of free choice as a content of perception/cognition can be conceivable as a result of strictly deterministic neuro-physiological sequence (in the Brain Time). This is analogous to the failure of one-to-one mapping in the temporal domain between the perceived sequence of two events and the underlying and corresponding neural events (section Underlying Neural Mechanisms? and Libet's Claims, and the “free will” Endangered?).
  As we noted, the perceived timing of an event should be considered separately from the physical timing of its neural correlates, particularly on the microscopic time scale (Nishida and Johnston, 2002). Thus, what is termed the “first-order isomorphism” may not hold between the perceived sequence and the physiological sequences of their neural correlates.
  Köhler's psychophysical isomorphism assumed that an organized structure of percept (such as relative sizes) has a direct counterpart in a common structure (relative sizes) of the dynamic neural field in the brain (Köhler, 1940). He used figural aftereffect as an example in the space domain. His claim has been criticized as being “too literally isomorphic,” and is thus sometimes called the “first-order isomorphism.” At present, neuroscientists do not believe that the neural correlates of “a figure A being perceived as larger than another figure B” should be “the neural circuit encoding A being spatially more extending than that encoding B.” Indeed, there are notable exceptions in which a larger stimulus naturally activate a larger cortical area (Murray et al., 2006; Schwarzkopf et al., 2011), but it is very limited to the early visual cortices where a strict retinotopic mapping is maintained.
  Because the skepticism on the first-order isomorphism is already a commonsense notion in the field, it is rather puzzling that the majority of scientists and philosophers still believe in such a first-order (direct) isomorphism in the time domain, between the temporal sequence of neural correlates and the time/sequence perception as the contents, especially on the microscopic scale.
  Similarly, a cognitive content (a feeling of agency, spontaneity or volition) can be considered separately from its neural correlates of it. To be more specific, a neural process may causally determine that a given action is felt voluntary or not (as the cognitive content), whereas that neural process remains to be entirely deterministic. This inevitably argues for involvement of postdictive and possibly semantic functions carried by the neural mechanisms subserving the higher-order perceptual experiences, with transformations of reality and illusions being typical for this symbolic level. Note also that being stochastic is categorically different from being voluntary; hence, the author would not endorse to some attempts to rely on the stochastic/undeterministic properties of neural dynamics to save free will and consciousness from determinism.
3. *A feeling of free choice is very much like a perceptual illusion, in that it will not be eliminated by objective knowledge*.
  Not all types of non-veridical perception are considered perceptual illusions in the “authentic” sense of psychophysics. For example, various sensory and cognitive hallucinations in the schizophrenia should not be considered perceptual illusions. Other than the fact that a percept is non-veridical with regard to the pertinent physical properties, a perceptual illusion should satisfy the following criteria, traditionally.
  (a) It should occur more or less similarly to the vast majority of neurotypicals. In other words, it should reflect normal, as opposed to pathological, sensory neural processing.
  (b) Objective knowledge (such as “the two lines are of equal lengths,” “the two disks have the same brightness of gray” etc.) typically will not eliminate this. Readers may go back to the flash lag, the “spatial memory updating (with the Duncker illusion), and many classical geometric illusions as qualified examples. This is presumably due to a modular structure of the sensory processing, that is free from top-down and the other cognitive modules at least partly.

Just as with perceptual illusions, the feeling of “agency” or “free choice” is unlikely to be “exorcised” by scientific knowledge of the underlying neural mechanisms (although actually no empirical data are available). This is similar to color perception in that the subjective color experiences (as some want to call “qualia”) would not disappear (as everyone's intuition tells) when color perception is fully *explained out* in neurophysiological terms, starting from photoreceptors, retinal ganglion cells, the LGN, through to the primary visual cortex, etc. And this is true even though color perception is also in a sense an “authentic illusion” because colors do not exist in the world, they are rather created by interactions between the physical stimuli and the brain. Likewise, the feeling of agency/free choice can be regarded as one type of robust, healthy and authentic illusion, for most of which not many people are concerned about the degree of compatibility to scientific determinism.

One may consider this view just as a variation of the “free will as a cognitive illusion” view proposed by Daniel Wegner and his colleagues (Wegner and Wheatley, 1999; Wegner, 2002). According to their view, people can experience conscious will quite independent of any actual causal connection between their thoughts and actions. The impression that a thought has caused an action rests on a causal inference.

Thus at a very crude level, the postdictive construction view shares a lot with Wegner's view of free will as a sort of cognitive illusion. Yet, there are several distinctive differences that would be noteworthy. Wegner's view has an obvious implication that free will is “an illusion, therefore wrong” with regard to the “true” physical causation. For instance, They make an analogy of the free will to a magic, in that there are real, and “disguised” causal relationship. The experience of conscious will in their view is merely an illusion produced by the perception of an apparent causal sequence. Apparent mental causation is generated by an interpretive process that is fundamentally separate from the mechanistic process of real causation.

Whereas we agreed that the free will (together with the sense of agency) is a mental construction, we take a somewhat different view. The free will reflects a normal function of the very general processing principle in the brain, i.e., postdictive construction employing the re-entry, the backward referral and possibly other mechanisms, which then leads to a normal experience of the “sense of agency.” In the very same sense as most of the geometric illusions qualify, it should rather be considered an authentic, or valid illusion based on mostly automatic, implicit processes.

Another deviation of our view from Wegner's “illusion” view is related to the three criteria they proposed for the interpretive process to experience free will. Those are (1) priority, (2) consistency, and (3) exclusivity. Among them we would like to impose a substantial constraint on the first criterion, i.e. the priority. As obvious from the detailed examination of various postdictive phenomena in the current article, starting from very sensory to highly cognitive levels, the priority may only be a distinctive feature of *the output* (i.e., the percept) of the processing, *not the physical condition* for it. As a matter of fact, all the three properties above, including the consistency and the exclusivity, may be, at least in some cases, results of postdictive reconstruction.

Whereas the two views are consistent in various aspects, this single contrast (priority vs. postdiction) may highlight the stark distinction.

## Summary and general discussion

This paper reviewed “postdictive” perceptual phenomena known, in which a stimulus presented later seems to causally affect percept of another stimulus presented earlier. Starting from some classical examples such as backward masking and apparent motion, the list included the cutaneous rabbit effect and the flash lag effect. Some new studies such as the TMS-triggered scotoma and the pursuit mislocalization suggest that various visual attributes are reorganized in a postdictive fashion to be consistent with each other, or to be consistent in a causality framework.

We then extended our discussion into several directions. First, in terms of underlying mechanisms, four prototypical models have been considered: the “catch up,” the “reentry,” the “different pathway,” and the “memory revision” models. Whereas they are meant to account for the “postdictive” phenomena but only in the operational sense above, the mechanism itself does not have to be postdictive in any sense (perhaps with the exception of the “reentry” model, and the “backward referral” idea by Benjamin Libet).

Second, by extending the list of postdictive phenomena to memory, sensory-motor and higher-level cognition (e.g., “hindsight”), one may note that such postdictive reconstruction may be a general principle of neural computation, ranged from milliseconds to months of time scale, from local neuronal interactions to long-range connectivity, in the complex brain. The operational definition of the “postdictive phenomenon” can be applicable to such sensory/cognitive effects across a wide range of time scale, even though the underlying neural implementations may vary across the variety of phenomena.

This notion of generic postdiction has a good affinity with the Bayesian framework, as well as the notion that perceptual awareness is in fact a very brief (possibly iconic) memory. As obvious in the case of a flicker perception previously mentioned (section Underlying Neural Mechanisms?), it is hard to draw a line between conscious perception and memory. And this is where a postdictive process operates on the preceding implicit process to yield a conscious visual percept.

Finally, this structurally the same mechanism may apply to body movements and its attribution to “free will.” The “sense of agency” which is the basis of “free will” may be considered a sort of “authentic illusion” which may hardly evaporate merely by reductionistic neural account for it.

Closer examinations of the postdictive phenomena may provide an entirely new and insightful framework to understand perception, cognition, memory and action. Moreover, it may add a new angle in the discussion of implicit vs. explicit mental processes, determinism vs. free will, etc.

### Conflict of interest statement

The author declares that the research was conducted in the absence of any commercial or financial relationships that could be construed as a potential conflict of interest.
